# Supplementary material for: Self-reported emotional and behavioral problems among school-going adolescents in Nepal—A cross-sectional study
Source: PLoS One. 2023 Jun 23;18(6):e0287305. doi: 10.1371/journal.pone.0287305 (PMC10289424; doi:10.1371/journal.pone.0287305)
Supplement: Attachment — Submitted filename: Response to Reviewers.docx [file pone.0287305.s002.docx]

**Response to the Reviewer’s comments**

Dear Reviewer,

We would like to thank you for reviewing our manuscript. Your comments and questions have been valuable to improve the quality of our paper. We have revised the manuscript according to your advice, to the best of our knowledge. References have been revised accordingly, and we have added some more references in the discussion section. According to the advice of the journal, we have provided a thorough language proof-reading of the whole manuscript to improve the language so that it will now be clear, correct, and unambiguous, and typographical and grammatical errors have been corrected. Below, we have given our answers to your comments one by one. In doing so, we have referred to the corresponding lines in our revised and proof-read manuscript with track changes (see *Revised manuscript with track changes*).

**Reviewer comment 1:**

“*The introductory section cites several prevalence estimates of EBP in the youth. In this section, it is important to present the validity of the measures used with respect to clinical diagnoses. Self- reports are general used as screening tool and generally tend to overestimate prevalence. This is important because the authors conclude that the results may be relevant for clinicians*”.

**Our Reply:**

Thank you for this comment. The introduction was written to give an overview of prevalence estimates of adolescents’ mental health problems in general populations as measured by screening instruments as this was the focus of our paper. However, we agree that prevalence obtained by screening instruments often differ from those obtained by diagnostic tools or -interviews. Hence, we have added the following sentences in the “Introduction” section:

“*One reason is that the prevalence may vary according to the measurement tools that are used in different studies. Generally, screening instruments may yield higher prevalence compared to diagnostic tools* *[10, 11].**”* See line number 72-74 in the revised manuscript.

In addition, we have added the following sentences about the validity of the tool that we have used (The Youth Self-Report – YSR) in the “Materials and Methods” section:

*“It has been found to have good content validity and criterion validity suggesting its ability to discriminate between clinical and non-clinical populations[53]. It can provide guidelines for the use of clinical cut-off points for various purposes.”* See line number 170-173 in the revised manuscript.

Finally, we added the following sentences in the “Discussion” section as follows:

*“It should be noted that the higher prevalence in our study might be due to methodological issues since we used a screening instrument (YSR). Studies using screening tools are likely to overestimate the prevalence rates compared to studies using validated diagnostic interviews or rates based on diagnostic criteria [10, 62].* See line number 330-333 in the revised manuscript.

**Reviewer comment 2:**

*“While the data presented in Table 3 shows that the clinical degree of EBP based on T-scores is higher among boys than girls for the Total, Internalizing and Externalizing categories, the authors rather chose to discuss only the results in Table 4. It would be more appropriate to discuss the findings in Table 3 or provide a good reason why the authors chose not to”.*

**Our Reply:**

Thank you for the comment. In Table 3, we calculated the prevalence estimates based on American norms (T-scores) and in Table 4, we presented the mean scores (raw scores). As the T-scores were based on American norms, we cannot say for sure that Nepali children’s problems were categorized according to relevant cut-offs. As we regarded the raw scores as more valid due to the unavailability of Nepali norms for the YSR, we decided to discuss our results mostly based on Table 4 and not to pay too much attention to the “clinical status” of the children. Further, by using the scale scores, we could keep information about each individual’s level of problems, while the classification into “normal”, “borderline” and “clinical” would make us lose some information. Lost information is yet another reason for not discussing the prevalence results into much detail. To clarify, we have now extended the discussion about internalizing versus externalizing problems in the “Discussion” section of the revised manuscript by adding the following explanation:

*“In our study, the prevalence of internalizing problems in boys was 28.7% compared to 19.3% for girls. This higher prevalence for boys should be regarded with caution as the prevalence estimates were based on American norms, and we cannot say for sure that Nepali children’s problems were categorized according to relevant cut-offs. Hence, we will focus more on differences in mean scores (raw scores) that are independent of norms.*” See line number 358-362 in the «Discussion» section of the revised manuscript.

We also extended the paragraph about internalizing versus externalizing problems in the “Discussion” section by adding the following about a recent American study:

*“Further, our findings are in line with a recent American study suggesting a trend of increasing internalizing symptoms and decreasing externalizing symptoms among adolescents during the last decennium [64].* See line number 338-340 in the revised manuscript.

**Reviewer comment 3:**

*“The discussion section emphasizes too much the cultural aspects of why externalizing symptoms are lower in prevalence. This is probably only one of the several reasons. Biological factors such as sex hormones especially among adolescents play an important role in regard to aggressive behaviors. The discussion should include the potential role of biological factors in the expression of symptoms from earlier publications”.*

**Our Reply:**

Thank you for this important input. We agree that we should have mentioned other explanations of behavior, such as biological factors. According to your advice, we have now added the potential role of biological factors and referred to earlier publications in the “Discussion” section:

*”However, cultural differences might be only one of several reasons for the differences in internalizing versus externalizing problems. Biological factors such as genes and sex hormones also impact the expression of symptoms, especially in adolescence [69, 70]. For instance, the male hormone, testosterone, is found to be associated with aggressive, rule breaking behaviors[71]. However, to examine the biological aspect of adolescent behaviors was beyond the scope of the present study”.* See line number 352-357 in the revised manuscript.
