# Supplementary material for: Self-reported emotional and behavioral problems among school-going adolescents in Nepal—A cross-sectional study
Source: PLoS One. 2023 Jun 23;18(6):e0287305. doi: 10.1371/journal.pone.0287305 (PMC10289424; doi:10.1371/journal.pone.0287305)
Supplement: Attachment — Submitted filename: Comment for PLOS ONE.docx [file pone.0287305.s003.docx]

I think this paper is an important addition to the child and adolescent psychiatry literature. Congratulations to all authors! However, I would like to recommend a few changes in all parts of the manuscript in terms of grammar/language, conciseness in writing, and the addition of a few pieces of information.

1. Though the author has mentioned thorough proofreading of language being carried out in response to the previous reviewer’s comments there are still typographical and grammar mistakes in the manuscript which need correction.

eg:

Line 120 “child competences”-should have been competencies.

In line 173 “TRF”-should have been “YSR”

There are still grammatical errors that need to be addressed like needs the addition of ‘the’; ‘for’; ‘a’; the need to change the word ‘from’ to ‘in’, and the correct plural/singular forms.

2. Regarding conciseness in writing for clear understanding;

- Lines 75-76 need revision;
- Lines 116-117 need correction;
- Lines 136-137 need to be re-written;
- Line 183 needs to be rewritten
- Lines 205-206 need revision,
- Lines 315-316 need revision.

3. Addition of information:

- Introduction 1^st^ paragraph needs elaboration on EBP and an explanation of how EBP is related to the mental health of adolescents. The manuscript seems to lack this point creating confusion between EBP and mental health problems as these terms are independently written.
- There is not much background information regarding adaptive functioning however, items related to adaptive functioning have been explored. Similarly, it has not been discussed much in the discussion section questioning its pre-requisite.
- Is it correct YRF has 105 items and TRF has a total of 128 items which has been mentioned in the manuscript?
- The manuscript has not mentioned copyright issues of both YRF and TRF. Neither has it been mentioned in the manuscript by Ma and colleagues.
- The description of YRF and TRF are very imprecise. What are the items related to adaptive functioning within TRF?
- Were the physical illnesses and life events assessed by only one question or there were further questions? Like duration for chronic illness and disabilities.
- What was the result of chronic illnesses and disabilities in terms of types?
- What was the result of serious life events in terms of types? Since the question was asked to parents, how do you justify its reliability, especially for traumas like sexual abuse?
- Why do you think the internal consistency of some item syndrome scales of YSR and the correlation between some sub-items-syndrome scales was weak?
- Line 235-236, how is the mention of the sample population to be in accordance with the overall Nepal population distribution justifiable when your sampling was purposive?
- Since YSR was not validated in Nepali and did not have the norms, I am interested to know what could have been the finding of EBP if the norms from a culturally similar country were applied!
- Did you explore the relationship between EBP and two broad-band scales? It would have been interesting to know if internalizing problems were common in adolescents suffering from severe traumas.
- Lastly, as you have explained the reasons for the increased prevalence of internalizing problems in adolescents our culture of discouraging verbal expression and emotional expression from childhood additionally might have played the role in the occurrence of internalizing problems.
